# Sulfate reduction controlled by organic matter availability in deep sediment cores from the saline, alkaline Lake Van (Eastern Anatolia, Turkey)

**DOI:** 10.3389/fmicb.2013.00209

**Published:** 2013-07-29

**Authors:** Clemens Glombitza, Mona Stockhecke, Carsten J. Schubert, Alexandra Vetter, Jens Kallmeyer

**Affiliations:** ^1^Geomicrobiology Group, Institute of Earth and Environmental Sciences, University of PotsdamPotsdam, Germany; ^2^Department of Surface Waters – Research and Management, Eawag, Swiss Federal Institute of Aquatic Science and TechnologyDubendorf, Switzerland; ^3^Department of Surface Waters – Research and Management, Eawag, Swiss Federal Institute of Aquatic Science and TechnologyKastanienbaum, Switzerland; ^4^Section 4.3 Organic Geochemistry, Helmholtz Centre Potsdam, GFZ German Research Centre for GeosciencesPotsdam, Germany

**Keywords:** saline lake, alkaline lake, sulfate reduction, deep biosphere, organic matter

## Abstract

As part of the International Continental Drilling Program deep lake drilling project *PaleoVan*, we investigated sulfate reduction (SR) in deep sediment cores of the saline, alkaline (salinity 21.4‰, alkalinity 155 m mEq^-1^, pH 9.81) Lake Van, Turkey. The cores were retrieved in the Northern Basin (NB) and at Ahlat Ridge (AR) and reached a maximum depth of 220 m. Additionally, 65–75 cm long gravity cores were taken at both sites. SR rates (SRR) were low (≤22 nmol cm^-3^ day^-1^) compared to lakes with higher salinity and alkalinity, indicating that salinity and alkalinity are not limiting SR in Lake Van. Both sites differ significantly in rates and depth distribution of SR. In NB, SRR are up to 10 times higher than at AR. SR could be detected down to 19 mblf (meters below lake floor) at NB and down to 13 mblf at AR. Although SRR were lower at AR than at NB, organic matter (OM) concentrations were higher. In contrast, dissolved OM in the pore water at AR contained more macromolecular OM and less low molecular weight OM. We thus suggest, that OM content alone cannot be used to infer microbial activity at Lake Van but that quality of OM has an important impact as well. These differences suggest that biogeochemical processes in lacustrine sediments are reacting very sensitively to small variations in geological, physical, or chemical parameters over relatively short distances.

## INTRODUCTION

**Figure 1:**
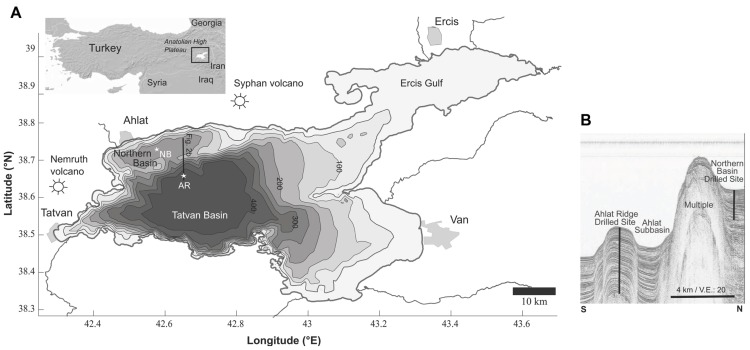
**(A)** Bathymetric map of Lake Van, showing the major basins, volcanoes and cities. The two drill sites are marked with stars (AR, Ahlat Ridge; NB, Northern Basin). **(B)** Seismic profile along a N–S transect, showing the basins and ridges. Note: the NB drill site is west of the seismic line is a projection and draw here for comparison. Modified from Litt et al. (2009).

Lake Van is located on the East Anatolian high plateau in southeast Turkey (**Figure 1**). The lake surface is currently at 1,674 masl. With a volume of 607 km^3^, a surface area of 3,570 km^2^ and a maximum depth of 445 m, Lake Van represents the fourth-largest terminal lake by volume (Litt et al., 2009) and the largest soda lake (Kadioglu et al., 1997) in the world. Filling a tectonic depression, the lake extends WSW-ENE for 130 km. In the northeast, the Eurasian Plate collides with the Afro-Arabian Plate resulting in an active fault system and causing hydrothermal activity, earthquakes and active volcanism (Degens et al., 1984). In the vicinity of Lake Van, there are two semi-active volcanoes Nemrut (3,050 masl) and Süphan (3,800 masl; Karaoglu et al., 2005). The latest eruption of Nemrut was recorded in 1,441 (Aydar et al., 2003). As a result of chemical weathering of volcanic rocks and evaporation processes, the lake water is saline (21.4‰) and alkaline (155 m mEq^-1^, pH 9.81; Kempe et al., 1991). Several changes in lake level were reported from previous investigations. Lake level high-stands of up to 90 m above the current level are documented by coastal outcrops (Landmann et al., 1996; Kuzucuoglu et al., 2010). Low-stands of up to -200 m below the present level, e.g., during the Younger Dryas, have been reported earlier (Lemcke, 1996; Litt et al., 2009).

In lakes and concomitantly in lake sediments, the sulfur cycle is an important element cycle, with sulfate reduction (SR) being a major electron acceptor process in anoxic sediments (Holmer and Storkholm, 2001). Various sulfate-reducing microorganisms are adapted to highly alkaline and saline environments (Oremland et al., 2000; Sorokin et al., 2008).

Most pore water sulfate originates from the overlying water, other minor sources are production of sulfate during hydrolysis of thioesters (King and Klug, 1982) and reoxidation of reduced sulfur species by various oxidants (Jørgensen, 1977,^1990^; Schippers and Jørgensen, 2002). Recently, Holmkvist et al. (2011) proposed that the pore water sulfate “background” of <0.5 mmol L^-1^ found below the sulfate methane transition zone (SMTZ) in sediment cores from Aarhus Bay (Denmark) is due to oxidation of H_2_S by iron minerals. By adding sulfate and organic substrates, they were able to stimulate SR and could thereby show that this sediment zone hosts a physiologically intact community of sulfate-reducing bacteria. They proposed that there is a “hidden sulfur cycle” that involves both sulfate generation and SR at low rates.

Another potential source for sulfate is the reoxidation of sulfide due to nitrate reduction (Laverman et al., 2012). However, in Lake Van this process does not play a quantitatively important role because nitrate concentrations throughout the water column are below 1 μmol L^-1^(Reimer et al., 2009) and the bottom water is anoxic (Kaden et al., 2010), so nitrate production due to ammonium oxidation in the sediment can be ruled out.

The majority of studies on SR have been carried out in marine sediments. Due to the high abundance of sulfate in seawater (28 mmol L^-1^) SR is the quantitatively most important terminal electron acceptor process for organic matter (OM) remineralization in marine sediments (Jørgensen, 1982). Sulfate concentrations in freshwater lakes usually range from 10 to >500 μmol L^-1^, significantly lower than in the marine realm, but there are some exceptions (review in Holmer and Storkholm, 2001). As a result of the low sulfate concentrations in most freshwater lakes sulfate usually penetrates only a few millimeter to tens of centimeters into the sediment (Smith and Klug, 1981). The most active SR is found within the top 10 cm. In alkaline soda lakes, sulfate concentration can sometimes reach up to several hundred mmol L^-1^, e.g., Searles Lake (California) with a sulfate concentration of 730 mmol L^-1^(Kulp et al., 2006). Sulfate concentration in Lake Van (20 mmol L^-1^) is relatively close to seawater.

In addition to salinity and sulfate concentration, important factors controlling the rates of SR are OM concentration and availability (Schubert et al., 2000). The OM acts as an electron donor for microbial metabolism and represents the redox partner for an electron acceptor like sulfate. Various studies show the importance of OM availability for SR in marine (Jørgensen, 1977; Niggemann et al., 2007) and lacustrine sediments (Blodau et al., 1998; Stam et al., 2010). In this context, the freshness or the state of degradation of sedimentary OM might play an important role. In recent studies it was shown that in marine sediments, microbial activity correlates rather with OM quality (indicated, e.g., by the content of chlorophyll pigments and their early degradation products like chlorins) and not with the bulk organic carbon content (Schubert et al., 2005; Niggemann et al., 2007).

While there is common agreement that the deep marine biosphere has a profound impact on global biogeochemical cycles (Jørgensen, 2012), much less attention has been paid to processes in deep lacustrine sediments, although lakes contain a significant fraction of the world’s usable drinking water (Downing et al., 2006) and are therefore of high societal relevance. Studies about the microbial abundance and activity in lacustrine sediments have so far only covered the upper centimeters to meters of sediments (Holmer and Storkholm, 2001). Also, biogeochemical studies on lake sediments described environments that differ considerably from Lake Van, for example “normal” (with regard to salinity and alkalinity) freshwater lakes, lakes affected by acid mine drainage (Blodau et al., 1998) or hypersaline environments like Mono Lake (Foti et al., 2007; Stam et al., 2010) or Lake Logipi (Castanier et al., 1993).

Geomicrobiological studies of deeper lake sediments are rare, mainly as a result of technical and logistical problems prevented the inclusion of geomicrobiological research in deep lake drilling projects. During the last decade, sampling equipment and analytical methods have improved substantially and paved the road for an increasing number of studies on deep lacustrine sediments (e.g., Vuillemin and Ariztegui, in press). Lake Van provides a high-resolution climate archive because the sediment is annually laminated (Wick et al., 2003) and intercepted by turbidites and ash layers of variable thickness. The high temporal resolution of the sedimentary record and its location in a climatically sensitive zone make Lake Van very attractive for paleoclimate studies and, thus, was chosen for the drilling project *PaleoVan* of the International Continental Drilling Program (ICDP; Litt et al., 2009,^2011^).

## MATERIALS AND METHODS

### SITE DESCRIPTION

Lake Van consists of two major basins and several smaller subbasins which are separated by basement highs and ridges (Utkucu, 2006; **Figure 1**). The Tatvan Basin is located in the center of the lake and represents the deepest and largest basin of Lake Van. The Northern Ridge separates the smaller and shallower Northern Basin (NB) from the Tatvan Basin. Ahlat Ridge (AR) is a small ridge bordering the small Ahlat Subbasin, which is located between NB and Tatvan Basin (**Figure 1B**; Litt et al., 2009).

Two drill sites were chosen for the ICDP drilling operation. The first site was located in the center of the NB at 260 mbll (meters below lake level) and the second site was located on top of the AR at 375 mbll (**Figure 1**). The Northern Ridge separates the two drill sites. The distance between the two sites is ~10 km.

### SAMPLING AND SAMPLE PROCESSING

Samples were obtained during the ICDP *PaleoVan* drilling campaign in summer 2010, using the Deep Lake Drilling System (DLDS) of DOSECC, Inc. The sediment cores reached a maximum depth of approximately 143 mblf (meter below lake floor; NB) and 220 mblf (AR; **Figure 1**). A hydraulic piston corer (HPC) was used for the upper approximately 100 m of sediment, for deeper sections a non-rotating (XN) as well as a rotating core bit (A) were used. During the drilling campaign, samples for immediate sample processing (pore water sulfate concentration and microbial turnover rate determination) were taken from the core catcher material. Core catcher samples obtained with the XN or A tool were often too disturbed to be used for microbiological or biogeochemical investigations as they were potentially contaminated by drilling fluid. Therefore, core catchers were only sampled if the sediment appeared to be undisturbed whole round pieces, which was the case for the majority of HPC core catcher material.

Small gravity cores of 65 to 75 cm length were taken at both drill sites to obtain the undisturbed sediment-water interface. Subsamples for SR rate (SRR) measurements, sedimentary OM characterization and pore water analysis were taken immediately after retrieval of the cores.

### PORE WATER SAMPLING

Sediment samples (~15 g wet weight) from the core catcher material of the drill cores were squeezed with an IODP-style titanium/PTFE pore water extraction system (Manheim, 1966) in a hydraulic press (2-column bench top laboratory press, 22 ton max load, Carver Inc., Wabash, IN, USA). The pore water was filtered through a 0.45 μm syringe filter and stored frozen until analysis. For the softer samples obtained from the gravity cores, approximately 10 g (wet weight) of the sediment was placed in a centrifuge vial and centrifuged for 10 min at 3,000 × *g*. The supernatant was collected, filtered through a 0.45 μm syringe filter and stored frozen until analysis. Prior to freezing, a few drops of pore water were used for measuring salinity with a temperature-compensated pocket refractometer (VWR, Darmstadt, Germany, Item No. 635-0171) and pH with a handheld pH meter (Metrohm) with a glass electrode.

### SULFATE REDUCTION RATE QUANTIFICATION

Sulfate reduction rates were quantified using the whole-core incubation method (Jørgensen, 1978) followed by cold chromium distillation (Kallmeyer et al., 2004). Samples were collected in duplicates using glass barrels with approximately 5 cm^3^ volume or 5 mL plastic syringes with cut-off tips. The glass barrels or syringes were used to retrieve small sub-cores from the undisturbed inner part of the core, sealed with a butyl rubber stopper and stored in sealed, nitrogen-flushed foil bags at 4^°^C prior to substrate incubation. Sediment from deeper sections of the core was highly compacted and very stiff, making it impossible to insert the thick-walled glass barrels. In such cases cut-off plastic syringes were used and stored in sealed, nitrogen-flushed foil bags.

Subsampling and storage of the SR samples in plastic syringes is not ideal even when storing the syringes in N_2_-flushed gas-tight bags due to the potential bias from gas exchange between the sample and the N_2_-atmosphere in the bag. However, in this particular case we assume that there is no major bias due to the following reasons. The sediment did not contain any major amounts of methane, and there is no indication from the SRR data that there was any SMTZ like in marine sediments. The loss of CO_2_ can also be neglected, given the pH of the pore water. Even if some minor amount of CO_2_ would be lost, there would not be any major shifts in pore water chemistry, given the extremely high buffer capacity.

Approximately 15 μL of ^35^S-labeled H_2_SO_4_ (1 MBq, Biotrend, Cologne, Germany) were injected along the axial center of the sub-core with a microliter syringe. The incubated samples were stored for 3 days at 4^°^C, which is the approximate in-situ temperature (Reimer et al., 2009). After the incubation period the sediment was transferred into 10 mL of 20% zinc acetate solution and frozen to trap all sulfide and terminate the incubation (Fossing and Jørgensen, 1989). Ten time-zero blank samples were prepared by injecting radiotracer into a sample, followed by transfer into zinc acetate solution after 10–15 min. Ten sediment-free tracer blanks were prepared by mixing 15 μL of radiotracer directly with zinc acetate. All blanks were processed like regular samples. Those twenty blank values, together with counter blank measurements (just zinc acetate and scintillation liquid) were used to calculate background and blanks according to Kallmeyer et al. (2004).

Samples were stored frozen until analysis. The ^35^S-labeled reduced inorganic sulfur (total reduced inorganic sulfur, TRIS) was extracted from the sample by the cold chromium distillation method of Kallmeyer et al. (2004). Radioactivity was quantified by liquid scintillation counting using Ultima Gold XR scintillation fluid (Perkin Elmer, Waltham, MA, USA) and a Packard 2500 TR liquid scintillation counter (Perkin Elmer, Waltham, MA, USA).

The SRR was calculated according to Eq. 1:

(1) $$\begin{array}{c} SRR\,=\;[{SO}_{4}^{2-}]\times P_{SED}\times \frac{a_{TRIS}}{a_{TOT}}\times \frac{1}{t}\times 1.06\times 1000, \end{array}$$

where SRR is the sulfate reduction rate, $\left[{SO}_{4}^{2-}\right]$ is the sulfate concentration in the pore water of the sediment sample (mmol L^-1^); *P*_ SED_ is the porosity of the sediment (ml pore water cm^-3^ sediment); *a*_TRIS_ is radioactivity of TRIS (total radioactivity of reduced sulfur species) [counts per minute (cpm) or decays per minute (dpm)]; *a*_TOT_ is total radioactivity used (cpm or dpm); *t* is incubation time in days; 1.06 is the correction factor for the expected isotopic fractionation (Jørgensen and Fenchel, 1974); 1,000 is the factor for the change of units from mmol L^-1^ to nmol cm^-3^.

### ION CHROMATOGRAPHY

For the quantification of chloride and sulfate concentrations, the pore water samples were diluted with deionized water 100- and 25-fold, respectively. Samples were analyzed in duplicates. The ion chromatography (IC) system was equipped with a LCA A14 column (SYKAM, Fürstenfeldbruck, Germany), a suppressor (SAMS^ TM^, SeQuant, Umeå, Sweden) and a SYKAM S3115 conductivity detector. The mobile phase was a 6.25 mmol L^-1^ Na_2_CO_3_ with 0.1 vol% modifier (1 g 4-hydroxy-benzonitrile in 50 mL methanol). Elution was performed at isocratic conditions. The eluent flow was set to 1 mL min^-1^. A blank sample and a standard solution containing 0.57 mmol L^-1^ chloride and 0.52 mmol L^-1^ sulfate were measured every 15 samples. Standard deviation of both standard and sample analysis was below 1% (determined from replicate analysis).

### TOTAL ORGANIC CARBON

Sediment samples of the gravity cores were stored at 4^°^C until analysis. The freeze-dried and homogenized samples were analyzed for total carbon (TC) and total nitrogen (TN) using an elemental analyzer (HEKAtech Euro EA, Wegberg, Germany). Total inorganic carbon (TIC) content was determined using a titration coulometer (5011 CO2-Coulometer, UIC Inc., Joliet, IL, USA). Total organic carbon (TOC) was calculated as TOC = TC - TIC and the C/N ratio as C/N = TOC/TN.

### DISSOLVED ORGANIC CARBON

In the 0.45 μm-filtered pore waters from NB and AR gravity cores, dissolved organic carbon (DOC) was quantified and characterized by size-exclusion-chromatography (Toyopearl-HW 50S resin, column size: 250 mm × 20 mm, Tosoh Bioscience, Stuttgart, Germany) with subsequent ultraviolet (UV;λ = 254 nm) and infrared (IR) detection in a liquid chromatography–organic carbon detection (LC–OCD) device (Huber et al., 2011).

Phosphate buffer 0.029 mol L^-1^; pH 6.5) was used as mobile phase with a flow rate of 1 mL min^-1^ (Huber et al., 2011). Quantification of DOC fractions by IR-detection of released CO_2_was achieved after UV photooxidation (λ = 185 nm) in a Gräntzel thin-film reactor (DOC Labor, Karlsruhe, Germany). Humic and fulvic acid standards of the Suwannee River (IHSS, International Humic Substances Society, St. Paul, MN, USA) were used for molecular weight calibration).

Dissolved organic carbon was separated into five fractions according to their molecular weight (MW): biopolymers (MW < 20,000 g mol^-1^); humic substances (~1,000 g mol^-1^); building blocks (300–500 g mol^-1^), which comprise mainly carbohydrates, phenols, and lignin monomers, lignin dimers, lipids, alkylaromatics (Schulten and Gleixner, 1999); low molecular weight (LMW) neutral compounds like alcohols, aldehydes, ketones (Sachse et al., 2001). LMW organic acids like acetate, propionate and other volatile fatty acids are rapidly consumed by microbes and represent the most readily available form of a carbon source (Finke et al., 2007).

### CHLORINS

The chlorin index (CI) and total chlorin concentrations were determined in gravity core samples according to (Schubert et al., 2005). In brief, 100 mg of freeze-dried and ground sediment was extracted three times with acetone under ice and in the dark. Extracts were measured with a Cary Eclipse fluorescence spectrophotometer (Agilent, Santa Clara, CA, USA). Excitation wavelength was 428 nm and emission wavelength was 671 nm. Chlorophyll a (Sigma) transformed to pheophytin by acidification with 100 μL of 25% hydrochloric acid was used as a standard. The CI is the ratio between the emission measured for the non-acidified and the acidified extract. The analytical precision of the method is ca. 5%.

### SEDIMENTOLOGICAL ANALYSIS

On site the drill cores were analyzed with a whole core multi-sensor core logger. The unopened cores were transported to the IODP core repository in Bremen, Germany, where they were opened, photographed and sampled. Lithologies from up to five parallel cores were correlated and a composite record from each drill site was constructed. The sediments were then categorized as either lacustrine sediments, fluvial deposits or volcaniclastic deposits. Next to the component-based classification, the sediments were subdivided into “background sediments” and “event deposits.” The background sediments (or pelagic sediments) cover all lithotypes reflecting the continuous, mostly environmentally controlled sedimentation of allochthonous and authochthonous material. The event deposits reflect instantaneously triggered deposition of allochthonous or reworked lacustrine material. The drill sites NB and AR were stratigraphically correlated by using laminated intervals and volcaniclastic layers. A chronology of the uppermost section was established by stratigraphic correlation to previously dated (varve chronology) sedimentary sequences of Lake Van (Landmann et al., 1996; Lemcke, 1996; Litt et al., 2009). A more detailed description of the individual measurements is given in (Stockhecke et al., unpublished data).

## RESULTS

### PORE WATER SULFATE, CHLORIDE, pH, AND SALINITY

**Figure 2:**
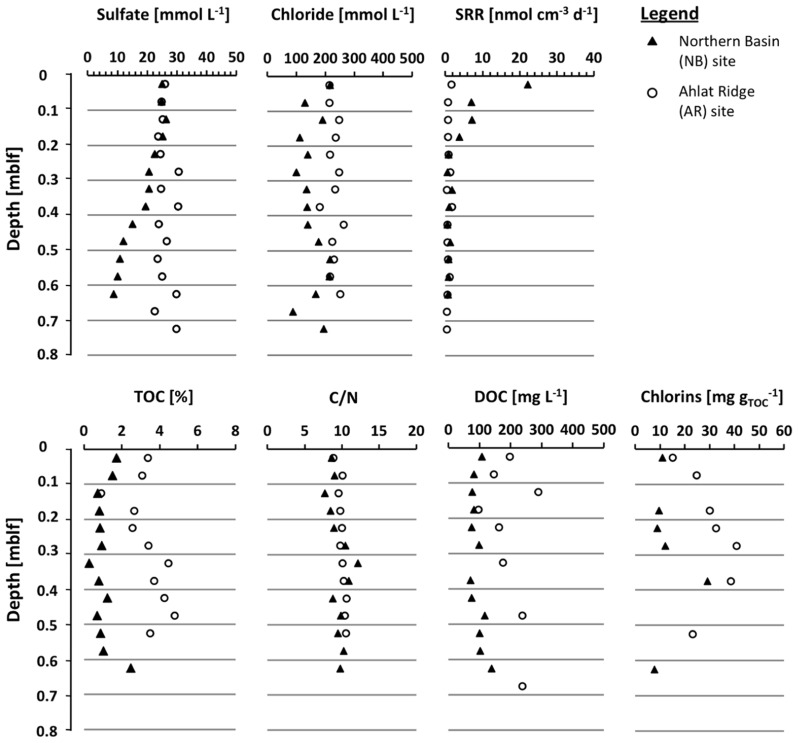
**Depth profiles of the gravity cores (0–0.75 mblf) from the Northern Basin site (NB, black triangles) and the Ahlat Ridge site (AR, open circles) for pore water concentration of chloride and sulfate, sulfate reduction rate (SRR), TOC concentrations, C/N ratio, DOC concentrations, and chlorins**.

**Figure 3:**
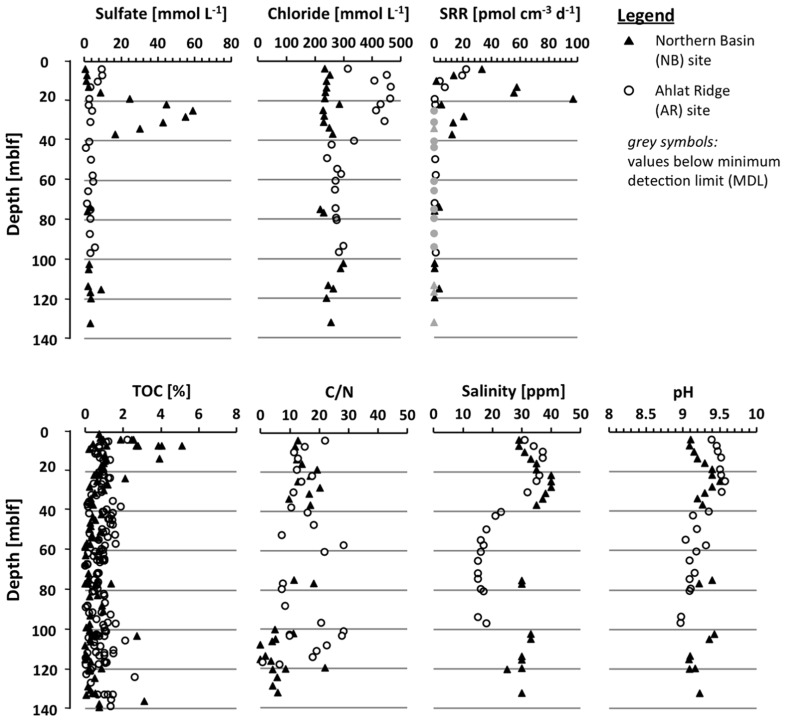
**Depth profiles of the drill cores (3.8–140 mblf) from the Northern Basin site (NB, black triangles) and the Ahlat Ridge site (AR, open circles) for pore water concentration of chloride and sulfate, sulfate reduction rate (SRR), TOC concentrations, C/N ratio, salinity, and pH of core catcher samples.** The light gray symbols in the SRR profile represent values below the minimum detection limit (MDL).

Porewater sulfate concentrations in the upper few cm of both sites are almost identical with 25 and 26 mmol L^-1^, respectively (**Figure 2**). In the pore water NB gravity core, sulfate concentration decreases with depth to 8.8 mmol L^-1^ at 0.65 mblf. Sulfate concentration in the AR gravity core shows some scatter but remains more or less constant throughout the core. The uppermost core catcher samples from drill cores from both sites were located at a depth of approximately 3.8 mblf, resulting in a lack of samples between ~0.7 and 3.8 mblf. The uppermost pore water sample from the NB drill core has a sulfate concentration of 0.5 mmol L^-1^ (**Figure 3**), indicating that sulfate was depleted by approximately 8 mmol L^-1^ over the missing ~3.1 m interval. At the AR site sulfate concentration in the lowermost gravity core sample and the uppermost drill core are 25 and 10 mmol L^-1^, respectively (**Figures2 and 3**). Sulfate concentration in the AR drill core decreases to values of 1–3 mmol L^-1^ at approximately 15 mblf, and remains constant throughout the rest of the core (**Figure 3**).

The pore water sulfate profile of the NB site shows a conspicuous concentration peak between 13 and 40 mblf with a local maximum of 59 mmol L^-1^ at 25 mblf (**Figure 3**). Below 40 mblf, sulfate concentrations remain at low values of 1–3 mmol L^-1^ at both sites. The pore water chloride profiles in both gravity cores (**Figure 2**) do not show much variation with depth and remain around 220 mmol L^-1^ over the upper 0.7 mblf. There is a slight excursion to lower values (~100 mmol L^-1^) at the NB site, but the lowermost and uppermost samples have almost the same concentration.

The chloride profiles in the drill cores (**Figure 3**) show a very different picture. At the NB site, pore water chloride concentration remains constant between 220 and 280 mmol L^-1^ throughout the core, whereas the AR site profile shows a pronounced positive excursion between 5 and 50 mblf with maximum concentrations around 480 mmol L^-1^. Below 40 mblf the pore water chloride concentrations are identical at both sites and remain between 220 and 280 mmol L^-1^.

Salinity and pH of the pore water samples (**Figure 3**) were measured on site. In the uppermost NB samples the pH is somewhat lower than at AR (9.1 vs. 9.4). At NB, pH increases to 9.5 around ~25 mblf before slightly decreasing again, whereas at AR, pH decreases below 30 mblf from 9.5 to ~9.0. At both sites salinity at the sediment-water interface is approximately 30 ppm. In the AR drill core it reaches a maximum of 37 ppm at 10 mblf whereas in the NB drill core a local maximum of 40 ppm is found at 25 mblf. Below 40 mblf salinity differs considerably between the two sites, with NB and AR around 25–30 and 15–20 ppm, respectively.

### SULFATE REDUCTION RATES

Maximum rates and depth distribution of SR differ significantly between both sites. At NB the maximum rates are about 5- to 10-fold higher than at AR (**Figure 2**). At both sites the main zone of SR is located in the upper few cm of the sediment. At NB the SRR show a decreasing trend, dropping from 22 nmol cm^-3^ day^-1^ in the uppermost sample (0–0.05 mblf) to approximately 1 nmol m^-3^ day^-1^ below 0.2 mblf. Values remain at this level throughout the rest of the gravity core (**Figure 2**). At AR, SRR are generally lower, with maximum rates of 2 nmol cm^-3^ day^-1^ in the uppermost (0–0.05 mblf) interval and values between 0.5 and 1 nmol cm^-3^ day^-1^ for the rest of the gravity core (**Figure 2**).

In deeper samples from the drill cores (**Figure 3**), low SRR in the range of single to tens of pmol cm^-3^ day^-1^ range were detected at both sites. At AR, SR is detectable down to 20 mblf with maximum values of 25 pmol cm^-3^ day^-1^. Below this depth SR above the minimum detection limit (MDL) could only be detected in a few scattered single samples. SRR in the NB drill cores initially follow the same trend like in the AR core. However, around 10 mblf, SRR start to increase again and reach up to 100 pmol cm^-3^ day^-1^ around 20 mblf before dropping below the minimum detection level around 40 mblf. Except for some single scattered samples, SR remained below the MDL throughout the rest of the NB drill core (**Figure 3**).

### TOTAL ORGANIC CARBON, CARBON-NITROGEN RATIO, AND CHLORINS

Total organic carbon content and carbon-nitrogen ratio (C/N) were determined on sediment samples of the gravity cores (**Figure 2**) and core catcher samples from both drill cores (**Figure 3**). On the drill core samples the amount of chlorins was quantified as well. At the NB site TOC is between 0.2 and 1.7%, showing a slightly decreasing trend over the upper 0.2 mblf. Deeper in the gravity core values scatter around 1%, except for the deepest NB gravity core sample which exhibits the highest TOC concentration in the entire core (**Figure 2**). In samples from the AR gravity core, TOC concentrations range between 2.5 and 4.8%, which is significantly higher compared to NB. After a slight decrease from about 4 to 2.5% over the upper 0.25 mblf values increase again and scatter around 4% throughout the deeper part of the core (**Figure 2**). In drill cores from both sites, TOC is more or less identical and scatters between 0.1 and 1.5%. In the upper 20 m of the NB drill core TOC shows more scatter with some values reaching up to 5% (**Figure 3**).

In both gravity cores C/N ratios are identical with values closely around 10, whereas in the drill cores C/N ratios show higher scatter at both sites. A decreasing trend with depth can be observed at both sites, although especially in deeper parts of the AR core there are broad variations (**Figure 3**).

Chlorin concentrations in the NB gravity core are low, showing a rather flat profile with values between 7.6 and 29 mg g_TOC_^-1^ (**Figure 3**). In the AR gravity core the chlorin concentration is higher, reaching values between 15 and 40.8 mg g_TOC_^-1^ with a maximum between 0.3 and 0.4 mblf. With the exception of the uppermost samples, the chlorin profiles resemble the TOC profiles in both gravity cores.

### DISSOLVED ORGANIC CARBON COMPOSITION

Dissolved organic carbon concentrations in the NB gravity core reveal a rather flat profile with values around 100 mg L^-1^ and a slight increase up to 150 mg L^-1^ in the deeper samples. In the AR gravity core the DOC concentrations are generally higher (100–280 mg L^-1^) but also show more scatter more than the NB gravity core samples (**Figure 2**). The high molecular weight biopolymer fraction was larger in samples from AR (61–161 mg L^-1^) than from NB (18–59 mg L^-1^). Concentrations of humic substances and building blocks were similar at both sites, in some cases with slightly higher values for AR. With the exception of the uppermost sample, the LMW neutral compound fraction at NB has a slightly higher concentration than at AR. Values decreased from ~40 to ~15 mg L^-1^ during the upper 0.4 mblf for NB and from ~28 to ~7 mg L^-1^ for AR. LMW acids were only detected in low concentrations in samples from NB and were absent in AR samples.

### SEDIMENT COMPOSITION

**Figure 4:**
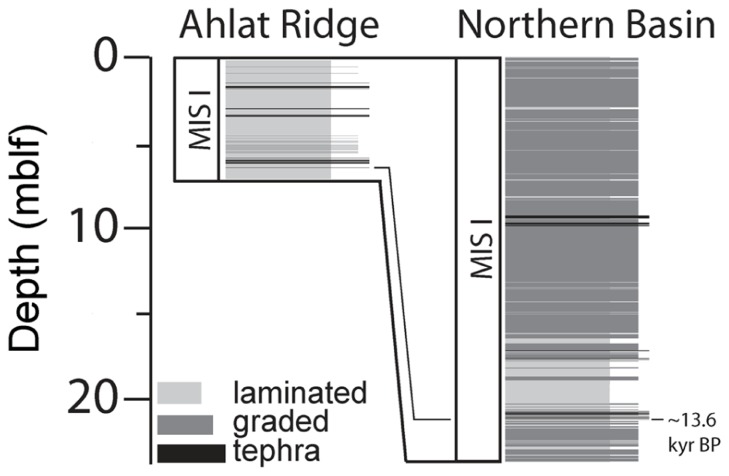
**Sedimentation at Northern Basin site and Ahlat Ridge site during the Marine Isotope Stage I (MIS I) including volcaniclastics, event deposits and lacustrine (laminated) sedimentation at NB and AR sites.** Especially sediments at the NB site are influenced by large amounts of graded event deposits and volcaniclastic deposits (tephra).

A detailed sedimentological analysis was carried out on the drill cores (Stockhecke et al., unpublished data). Drill site correlation revealed that sediment input in the upper ~20 mblf is approximately three times higher at NB than at AR and mostly related to the accumulation of thick event deposits, consisting of redeposited volcaniclastic and terrigenous material (**Figure 4**). These organic carbon-poor deposits largely influence sedimentation rates. Total sediment thickness since the onset of the YD is 20.6 m at NB and 5.74 m at AR (Stockhecke et al., unpublished data). After subtraction of the volcaniclastic deposits and event layers, sediment thickness changes dramatically at NB, leaving only 4.99 m of lacustrine sediment, whereas at AR, sediment thickness is only slightly reduced to 5.53 m.

## DISCUSSION

In this study, we conducted biogeochemical investigations in high resolution close to the sediment-water interface and in ~ 3-m resolution for deep subsurface sediments down to 130 mblf.

Holmer and Storkholm (2001) reviewed SRR data from several lake sediments, including eutrophic and oligotrophic lakes. For lakes with sulfate concentrations >1 mmol L^-1^ they report SRR in the range of up to several 100 nmol cm^-3^ day^-1^, which is significantly higher than the 22 nmol cm^-3^ day^-1^ that were found in Lake Van sediments. Also compared with other extremely alkaline and saline lakes, SRR in Lake Van sediments are generally very low. Besides the differences in SRR compared to other investigated soda lakes, both Lake Van drill sites differ significantly regarding maximum rates and depth distribution of SR, not just in the gravity cores but also in the drill cores. The uppermost drill core samples still show measurable SRR in the pmol cm^-3^ day^-1^ range. According to lithological investigation of the drill cores, the lowermost sample with detectable SR at the NB site corresponds to the end of the Younger Dryas and the beginning of the Holocene (~11.7 ka BP). As a result of different sedimentation rates between both sites, the lowermost sample with detectable SR at AR corresponds to Marine Isotope Stage 2 (MIS 2, ~28 ka BP). It is interesting to note that despite the generally low SR activity, SR can be detected down to 19 mblf (NB drill core), a depth range that has not been covered before by any microbiological investigations of lacustrine sediment. Despite the relatively small differences with regard to water depth and distance to shore and the relatively short distance between the sites (especially when compared to many marine transects), there is a clear difference in SRR. This suggests that biogeochemical processes in lacustrine sediments react very sensitively to variations in geological, physical or chemical parameters over relatively short distances.

### ALKALINITY AND SALINITY

Several studies investigated SR in shallow lake sediments and found SRR mainly in the range of hundreds of nmol cm^-3^ day^-1^, even in oligotrophic lakes (review in (Holmer and Storkholm, 2001). Most studies about the effects of high alkalinity on SR were carried out in lakes that are hypersaline or had at least a salinity more than twice as high as Lake Van, such as soda lakes from Kulunda steppe (Foti et al., 2007), Mono Lake (Oremland et al., 1993; Stam et al., 2010), Searles Lake (Kulp et al., 2006,^2007^), Soap Lake and Big Soda Lake (Oremland and Miller, 1993). These studies documented that microbial SR does occur in these extreme environments. However, several studies did not use the whole core incubation technique like we did, which is considered to provide the most realistic results (Jørgensen, 1978), but sediment slurries (Kulp et al., 2007) or flow-through reactors (Stam et al., 2010) instead. Both methods tend to overestimate rates, so the actual rates cannot be compared due to the different incubation techniques. In Mono Lake Kulp et al. (2007) measured highest SRR (660 μmol cm^-3^ day^-1^) at the site with the lowest salinity (25 g L^-1^). Despite being in a similar salinity range like Lake Van (~20 g L^-1^), these rates are over 20,000 times higher than the highest rates we measured. Although lower than the values reported by Kulp et al. (2007), the SRR measured by (Stam et al., 2010) in flow-through reactors containing intact sediment cores from Mono Lake are still up to 100 times higher than our measurements from Lake Van.

Foti et al. (2007) also used a whole core incubation to measure SRR in sediments of soda brine lakes from Kulunda Steppe, western Siberia. Even the most hypersaline lakes (e.g., Lake Tanatar, salinity: 475 g L^-1^, pH 10.6 and Picturesque Lake, salinity: 405 g L^-1^, pH 9.95) exhibit SRR that are higher (up to 423 nmol cm^-3^ day^-1^) than Lake Van sediments.

The studies of Oremland (Oremland and Miller, 1993; Oremland et al., 1993) both employed the whole core incubation technique on pelagic cores from Mono, Soap and Big Soda Lake. Highest SRR were always found in the upper 0.1 mblf. In some cases maximum SRR were roughly in the same range as in Lake Van sediments, but mostly they were up to 10 times higher.

This indicates that even when compared to lakes with much more alkaline and/or saline conditions, SRR in Lake Van are relatively low. Thus, alkalinity and salinity do not seem to limit microbial activity in Lake Van.

### SULFATE AVAILABILITY

The sulfate concentrations found in the uppermost gravity core samples at both sites are very similar to lake water concentrations of ~25.4 mmol L^-1^ measured in 400 m water depth in the Tatvan Basin (Reimer et al., 2009). These values are close to marine sulfate concentrations (28 mmol L^-1^). An obvious explanation for the strong increase in sulfate concentration in the NB drill core between 13 and 40 mblf (**Figure 3**) would be a lake level low-stand as lake level variations up to several hundred meters were reported (Landmann et al., 1996; Litt et al., 2009; Kuzucuoglu et al., 2010). During a lake level low-stand, caused by enhanced evaporation and decreased precipitation, the concentrations of all ions including sulfate is expected to be higher. The maximum sulfate concentration at ~25 mblf in the NB drill core coincides with the Late Glacial (~15 ka). However, in this case it is rather unlikely that the peak is simply a result of higher salinity during a lake level low-stand at the time interval of deposition, because the chloride concentration profile (**Figure 3**) does not show a similar excursion and remains flat throughout this interval. Although chloride has a higher diffusion coefficient than sulfate (1.12 × 10^-9^ m^2^ s^-2^ vs. 5.72 × 10^-10^ m^2^ s^-2^, at 5^°^C in seawater, respectively (Jøregensen, 2000) the sharp sulfate peak and the completely flat chloride profile rule out that the chloride peak was removed by diffusion. There is general agreement that the lake level dropped during the Late Glacial (Landmann et al., 1996; Kuzucuoglu et al., 2010). Additionally, the sedimentary solid phase sulfur content does not change over this depth interval, ruling out mineral dissolution as a source of sulfate. Another possible source of sulfate would be volcanic degassing, there are some indications for sublimnic volcanism in the Lake Van area (U. Schmincke, personal communication). A much more detailed geophysical and geochemical study will be necessary to elucidate the origin of this sulfate peak. Nevertheless, the fact that pore water sulfate concentrations show such large differences between the two sites might suggest that Northern and Tatvan Basin were disconnected or at least mixing processes had been suppressed during that time period, which is also suggested by facies analysis (Stockhecke et al., unpublished data).

### SEDIMENTARY ORGANIC MATTER CONTENT

The content of OM in the sediment plays an important role in controlling SR, since an increase of OM concentration will generally increase microbial activity (Jørgensen, 1977,^1982^; Canfield, 1989). The concentration of chlorins can be used to assess productivity of pigment-related OM in marine but also in lacustrine sediments (Meckler et al., 2004; Schubert et al., 2005; Naeher et al., 2012). Surprisingly, SRR are higher at NB than at AR although the chlorin and TOC as well as pore water DOC concentrations are lower. Thus, in Lake Van sediments, OM content does not directly correlate to SR activity, which indicates that OM concentration in the sediments alone cannot be used to infer microbial activity at Lake Van.

Due to its closer proximity to the shore and its location inside a basin, the NB site receives higher input of terrigenous material, which dilutes the lacustrine sedimentation. However, since the C/N ratios of both sites are very similar, the terrigenous input into NB does not result in higher amounts of terrestrially derived OM in the sediments. One possible explanation could be the sparse vegetation in the Lake Van area. However, event deposits at NB are more frequent and much thicker compared to the AR site (Stockhecke et al., unpublished data).

The organic carbon-poor volcaniclastics and event layers dilute the originally organic carbon-rich lacustrine sediment at NB about fourfold. Due to erosion at the base of the event layers in NB, the lacustrine, laminated sediments are even slightly thicker at AR. TOC values at both sites are around 1.5% but show considerable scatter. Especially in the top ~20 mblf at NB, values can reach up to 5% TOC, representing samples of purely laminated, event-free sediment with less diluted OM. The accumulation rates for OM, TOC and chlorin are suggested to be higher at NB than at AR due to a higher flux of bioavailable OM into NB sediments, thereby supporting microbial activity.

### SEDIMENTARY ORGANIC MATTER AVAILABILITY

An important factor controlling the bioavailability of sedimentary OM is its quality or, in other words, its reactivity or status of degradation (Lomstein et al., 1989; Niggemann et al., 2007). A higher degree of OM degradation will result in OM that is most likely more resistant to microbial utilization. In contrast, the availability of less degraded, reactive OM such as volatile organic acids is suggested to increase microbial activity. For example Blodau et al. (1998) found only low SRR in OM-depleted acidic lakes. DOC ranged from 6 mg L^-1^ in the lake water to 80 mg L^-1^ in the sediment pore water, which had moderate sulfate concentrations (2–6 mmol L^-1^). They were able to stimulate SR by addition of acetate to the sediment samples. Recently, Stam et al. (2010) reported similar findings from Mono Lake. Although sediments there have a high sulfate concentration (~100 mmol L^-1^) and high SRR and the experiments were performed in flow-through reactors, the addition of acetate to the sediment samples lead to a fivefold increase in SRR. Increased SRR were also reported for lactate additions to different freshwater, brackish and marine sediments (Pallud and Van Cappellen, 2006). These findings stress the role of easily degradable organic substrates on SSR.

A parameter indicating ongoing OM alteration during the very early stages of diagenesis is the CI (Meckler et al., 2004; Schubert et al., 2005; Carstens et al., 2012). This index describes the freshness of pigment-related compounds. Whereas fresh chlorophyll has a CI of 0.2, very degraded OM shows values around 1. An increasing CI reflects increasing diagenetic alteration of the organic material. However, CI values determined for Lake Van sediments showed almost no variations with depth for both sites and nearly all values were between 1.0 and 1.3. These high values have also been observed in other lacustrine systems (Schubert, unpublished results) and are so far not understood, making the CI not suitable for Lake Van sediments.

**Figure 5:**
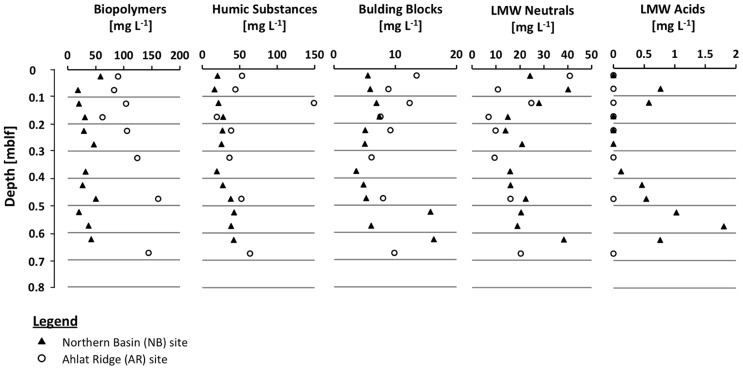
**Profiles of dissolved organic carbon (DOC) fractions in the pore water of both gravity cores including biopolymers (**<**20,000 g mol^-1^), humic substances (**~**1,000 g mol^-**1**^), building blocks (fragments of humic substances, 300–500 g mol^-1^), low molecular weight (LMW) neutrals (**<**350 g mol^-1^) and low molecular weight (LMW) acids (**<**350 g mol^-1^)**.

By having ruled out other factors such as salinity, alkalinity, sulfate and TOC concentration, we assume that OM quality plays an important role in controlling SRR in Lake Van even if the CI does not reflect this. When separating the pore water DOC of the two gravity cores into different size fractions, we found remarkable differences between the two sites. There is a higher percentage of LMW compounds at NB than at AR (**Table 1**). The depth profiles of the different DOC fractions show that the main difference in the total amount of DOC is mostly due to the biopolymer fraction, containing rather recalcitrant high molecular weight organic macromolecules >20,000 g mol^-1^ (**Figure 5**). The slightly higher percentage of humic substances and building blocks (smaller units of humic substances, ~1,000 g mol^-1^) in NB samples (38.5%) as compared to AR (34.4%) shows that the NB pore water contains only slightly higher amounts of terrestrial OM (**Table 1**). This is consistent with the almost identical C/N atomic ratios (**Figure 2**). Differences between the two sites were found in the amounts and compositions of the LMW fraction (<350 g mol^-1^). Samples from NB have higher amounts of LMW compounds than AR (25% vs. 9%). Moreover, LMW acids, which are prime microbial substrates, were only found in NB samples (**Figure 5**; **Table 1**). When looking at the pore water DOC composition of both sites (**Table 1**) it becomes obvious that the DOC in AR pore water is dominated by high molecular weight OM (biopolymers, humic substances and building blocks) accounting in total for 91% of the DOC, whereas LMW OM accounts for only 9% in AR pore water. In contrast, the HMW fraction of pore water DOC samples from NB accounts for only 75%, but the LMW fraction, which is considered a potential substrate for microbes (Glombitza et al., 2009), accounts for 25%, including 0.6% LMW acids which are absent at AR (**Table 1**). We thus suggest that availability of labile OM in the pore water plays a more important role in controlling microbial activity in Lake Van than sedimentary OM content.

**Table 1:** Pore water DOC composition of Northern Basin and Ahlat Ridge gravity cores (sum of all samples), including the individual fractions obtained in LC–OCD.

	Northern Basin (in % of DOC)	Ahlat Ridge (in%of DOC)
**Compound fraction**		
Biopolymers (>20,000 g mol-1)	36.5	56.6
Humic substances (-1,000 g mol-1)	30.7	29.5
Building blocks (300–500 g mol-1)	7.8	4.9
LMW neutral (<350 g mol-1)	24.4	9.0
**Grouped fractions**		
Macromolecular OM fraction	75	91
LMW fraction	25	9
Terrestrial OM fraction	38.5	34.4

*See main text for a detailed description of the different fractions.*

Studies by Niggemann et al. (2007) and Schubert et al. (2000) showed similar effects in the upwelling area off Chile. There, OM input into the sediment and quality of sedimentary OM decreased with increasing water depth and distance to shore. Compared to Lake Van, the sites off Chile were similar in distance from each other, but showed much greater differences in water depth (individual sites range between 126 and 1,350 m water depth; Niggemann et al., 2007). Compared to marine systems the 115 m difference in water depth between the two Lake Van sites is relatively small. Still there are significant differences in microbial activity. A possible interpretation could be that water depth has a much larger effect in lacustrine than in marine systems. However, more important for OM degradation might be the boundary between oxic and anoxic water (chemocline). It was shown in continental margin sediments that an oxygenated water column enhances OM degradation (Hartnett et al., 1998). In 2009, the chemocline in the Tatvan Basin was recorded at 250 mbll (Stockhecke et al., 2012). However, in 2005 oxygen was found down to 325 mbll in deep parts of the Tatvan Basin (Kaden et al., 2010). Just 15 years earlier, in 1990, oxygen was found even deeper at >400 mbll (Kipfer et al., 1994). In 2005, the chemocline in the shallower NB was found at 200 mblf. This suggests that in the shallower NB the layer of oxic surface water was generally thinner than in the deeper Tatvan Basin. Furthermore, especially during times of lake level low-stand, exchange processes between NB and Tatvan Basin might have been suppressed. The differences between the two sites with regard to pore water chemistry also support the assumption that the two basins were at least partially separated during their geologic history. During lake level low-stands a fully or partially isolated small NB might have experienced longer periods of bottom water anoxia reaching quite high up in the water column. Thus, sediments in the NB contain higher amounts of less altered OM than AR sediments. The thicker layer of oxic water at AR might account for the higher degree of OM alteration and concomitantly for lower microbial activity.

## CONCLUSION

In the scope of the ICDP *PaleoVan* project, we investigated SR in sediments of the alkaline soda lake Lake Van (Eastern Anatolia, Turkey). In comparison with other soda lakes SRR in Lake Van sediments were generally low (max. 22 nmol cm^-3^ day^-1^), with the uppermost sediments of the shallower NB site revealing SSR that are up to 10-fold higher than in AR sediments. SR is detectable down to depths of 19 mblf (~11.7 ka BP), whereas at AR the SRR fell below the MDL at depths >13 mblf (~28 ka BP). The differences in water depth and distance to shore between the two sites are relatively small compared to similar studies in marine environments, still the two sites in Lake Van differ in microbial activity. These differences suggest that lacustrine sediments represent highly sensitive study sites, which exhibit significant biogeochemical changes over relatively short distances according to changes in environmental conditions.

The low SRR in Lake Van sediments compared with other saline soda lakes indicate that salinity and alkalinity are not the limiting factors for SRR in Lake Van. Sulfate concentration can also be ruled out as a limiting factor because its concentrations always remains >1 mmol L^-1^. Sedimentary TOC and pore water DOC concentrations are higher at AR than at NB as a result of large event deposits and the input of terrigenous material, which both have a very low OM content. The C/N ratio in sediment samples and the concentrations of humic substances in the pore water from both sites are similar, indicating the same type of OM input in the sediment. Due to the sparse vegetation in the Lake Van area, NB does not receive larger amounts of terrigenous OM input. Chlorin concentrations resemble the TOC profiles in both sites. However, microbial activity is higher at NB than at AR. This shows that OM concentrations alone do not control the rates of SR in Lake Van sediments. Separation into size fractions revealed that the DOC at NB contains greater amounts of LMW compounds. Also organic acids, which are prime microbial substrates, were found only at NB. We thus speculate that OM quality and bioavailability is an important factor controlling SRR in Lake Van. At AR, the oxic-anoxic chemocline is deeper than at NB, resulting in stronger OM degradation in the water column and leading to more recalcitrant sedimentary OM. We suggest that OM in NB sediments is more available for microbial utilization than at AR, resulting in higher microbial activity.

## Conflict of Interest Statement

The authors declare that the research was conducted in the absence of any commercial or financial relationships that could be construed as a potential conflict of interest.
